# Commentary on a method for testing resistance to shocks

**DOI:** 10.1016/j.mex.2020.101194

**Published:** 2020-12-29

**Authors:** Paresh Kumar Narayan

**Affiliations:** Centre for Financial Econometrics, Faculty of Business and Law, Deakin University, 221 Burwood Highway, Burwood, Victoria 3125, Australia

**Keywords:** Coronavirus, COVID-19, Crude oil prices, Exchange rates, Narayan–Popp test, Persistency, Shock resistance, Time series, Unit roots

## Abstract

This note tours the Narayan (2020a: Has COVID-19 Changed Exchange Rate Resistance to Shocks?) approach to testing for resistance of a time-series variable to shocks. We take a step-by-step account of this approach and demonstrate its applicability with respect to the crude oil price.

• *The approach entails steps (1) to (8), as outline in the paper.*
• *Future researchers will find this method useful in evaluating the resistance of variables to not only COVID-19 shocks but to any shock which has had a sufficiently long life.*

## Graphical abstract

**Figure:**
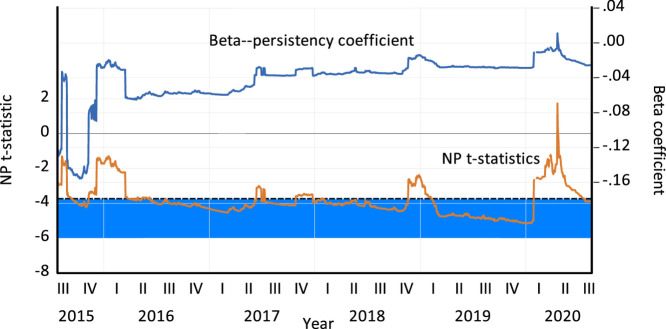

Specifications table

**Table:** 

Subject Area	Economics and Finance
More specific subject area	*Time Series Economics and Finance*
Method name	*Narayan–Popp (2010) test*
Name and reference of original method	*Narayan, P. K., & Popp, S. (2010). A new unit root test with two structural breaks in level and slope at unknown time. Journal of Applied Statistics, 37, 1425–1438.*https://doi.org/10.1080/02664760903039883.
Resource availability	• *Any time-series data* • *Available in Gauss Software*

## Introduction

A proliferation of studies on the COVID-19 pandemic has ignited the importance of shock resistance [10,^12^,^17^,^18^,^24^,^27^,^31^,^35^,^36^,^39^,37].^1^ There are several important facts of note with this pandemic. First, this is a lasting pandemic. It has come in phases, often referred to as “waves” by the medical profession. Therefore, the pandemic is volatile. It is longest in terms of a shock to the global economic and financial system [6,^29^,^38^,40]. It is unlike what we have seen, for instance, with financial crises. Second, the pandemic is still unfolding, reflecting the fact that the shock is persistent. Third, the manner in which COVID-19 impacts the economic and financial system is not homogenous—some sectors and indeed components of the economic system are affected more than others; see Narayan, Gong and Ahmed [25]; He, Sun, Zhang and Li [15]; and He, Niu, Sun, and Li [14]. This implies that some industries are more resistant than others.

The overall message is that the investigation of the resilience of the financial and economic system has not progressed much and this is perhaps one area of research on COVID-19 that will attract greater interest with time. In light of this, in a recent paper Narayan [26] propose a shock resistance methodology. We believe the applicability of this approach to understanding the response and indeed resistance of financial and macroeconomic time-series data is important for future researchers.

In this note, we go step-by-step covering the implementation of this approach and apply it to West Texas Intermediate crude oil data.

In the next section, we set out the step-by-step approach. In Section III, we present an application. We close with a remark on directions for future research.

## Methods details

### Step-by-Step method/approach

The Narayan [26] resistance methodology is in turn based on the Narayan and Popp [23], see also Narayan and Popp [22], unit root (NP-UR) test.

Step 1: Implement the NP-UR model that allows for two endogenous breaks in the level. There are no breaks in the trend.

Step 2: Choose an appropriate trimming factor to account for tails of the data. Typically, a 10%-20% trimming factor is employed depending on the sample size.

Step 3: Choose the initial data sample window to generate time-varying β. Narayan (2020) recommend a window size of 20%. Alternative window sizes may be motivated based on sample size.

Step 4: Run the following regression model using ordinary least squares:

(1) $$OIL_{t}=\alpha +\beta OIL_{t-1}+\lambda t+\kappa_{1}BR_{B,1}^{\prime }+\kappa_{2}BR_{B,2}^{\prime }+\delta_{1}DU_{1,t-1}^{\prime }+\delta_{2}DU_{2,t-1}^{\prime }+\sum_{j=1}^{k}\pi_{j}\Delta OIL_{t-j}+e_{t}$$

The variable which we consider for the test of resistance is the WTI crude oil price (OIL).

Step 5: Choose the lag length, k, which should be set high enough to accommodate serial correlation. When done, use one of the lag length selection criteria to select the optimal lag length.

Step 6: Obtain the $BR_{B,1}\;$and $BR_{B,2}$ which denote, respectively, the first and second break dates; and $DU_{1}$ and $DU_{2}$ are the level break dummy variables.

0Step 7: Test the null hypothesis of a unit root by setting β=1 against the alternative hypothesis that β<1.

Step 8: Use the critical values from NP [23] to decide on the rejection or otherwise of the unit root null hypothesis.

## Application to oil price data

In the application to the resistance methodology, we choose to focus on energy data because COVID-19 related studies on energy has become an important subset of the literature (see [9,^16^,^20^,^28^,^30^,^33^,34]). An important feature of this literature that inspires our application is the manner in which these studies treat COVID-19 vis-à-vis the economic and financial system. These studies, in other words, tend to ask how this COVID-19 shock is related to oil price [7,^11^,^16^,32]; US oil and gas producers [19]; oil price news [28]; stock markets [20]; corporate performance [9]; energy firms [30]; and diesel fuel volatility [8]. In other words, these studies test the response of energy related variables to COVID-19. While these studies use bivariate/multivariate models to evaluate responsiveness, we propose a univariate treatment of the variable's response directly.

**Fig. 1:**
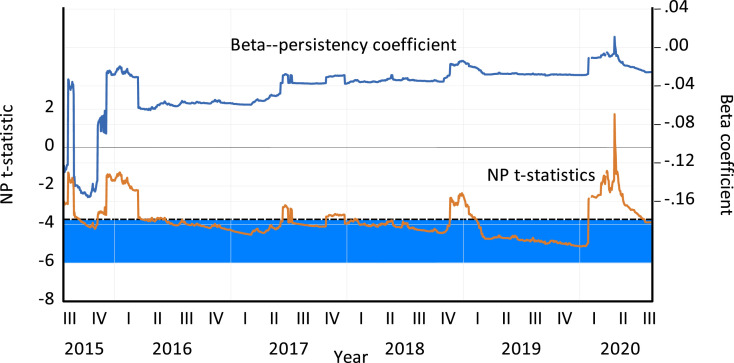
Time-varying shock persistence of Yen-US dollar exchange rate. This figure plots the time-varying beta based on the Narayan and Popp [23] two endogenous break unit root model. The 10% critical value (CV) of the NP test is −3.772 and is marked on the graph in dotted lines. The regression is estimated through expanding windows with the initial window set to be 10% of the sample data, which is 1/1/2015 to 8/17/2020 culminating into a total sample size of 1468 observations. This window is expanded by one observation post each estimation. We also have to identify a suitable trimming region. We set this to 10% of sample given our relatively small sample size. The area shaded in colour blue depicts the phases in which the unit root null was rejected suggesting that the shock did not have a permanent effect on oil price.

We use daily time-series hourly data on oil prices starting from 1/1/2015 to 8/17/2020 for a total sample size of 1468 observations. We make the following decisions for implementing the model: a maximum lag length of 8 is set to control for serial correlation; a trimming factor of 10% is imposed given our small sample size; a initial estimation window equivalent to 10% of the sample size is selected; and the window is expanded by one observation post each estimation. The time-varying beta and its *t*-statistic, where the 10% critical value is −3.77, are plotted in Fig. 1. The result is a total of 1320 estimation windows. Out of this, in 493 windows the unit root null hypothesis is rejected, suggesting that shocks to oil prices are transitory. In other words, in 37% of the windows oil prices are resistant to shocks. When we observe closely the response of oil prices in the most recent period, from 1 January 2020, we find 164 windows out of which the unit root null is only rejected in 61 windows. This implies that in over 60% of the windows the unit root null was not rejected, suggesting that the oil price has been less resistant to the COVID-19 shock.

## Concluding remark

This note connects to Narayan [26] where a method for testing the resistance or persistence of exchange rate to COVID-19 was developed. Using the Narayan and Popp structural break unit root test, framed on a rolling window setup, he extracts time-varying persistency parameter. This parameter is used to judge persistency of the shock. In this note, we provide a step-by-step guide to implementing this persistency test to oil price data. Future researchers will be able to utilize this test to examine the effects of COVID-19 or any shock for that matter on any macroeconomic or financial variable that have sufficient time-series data.

## Declaration of Competing Interest

The author declares that he has no known competing financial interests or personal relationships that could have appeared to influence the work reported in this paper.

## Footnotes

1 Others papers have considered COVID-19 and global trade [41]; COVID-19 and bitcoin [3, 4]; air quality [21]; insurance market [42]; household financial decision [44]; and labor force participation [43]. See also earlier papers on COVID-19: Al-Awadhi et al. [1]; Ali et al. [2]; Corbet et al. [5]; Haroon & Rizvi [13]; Zhang et al. [46]; Zaremba et al. [45].
